# An O-Antigen Glycoconjugate Vaccine Produced Using Protein Glycan Coupling Technology Is Protective in an Inhalational Rat Model of Tularemia

**DOI:** 10.1155/2018/8087916

**Published:** 2018-11-29

**Authors:** Laura E. Marshall, Michelle Nelson, Carwyn H. Davies, Adam O. Whelan, Dominic C. Jenner, Madeleine G. Moule, Carmen Denman, Jon Cuccui, Timothy P. Atkins, Brendan W. Wren, Joann L. Prior

**Affiliations:** ^1^Defence Science and Technology Laboratory, Porton Down, Salisbury, Wiltshire SP4 0JQ, UK; ^2^Department of Pathogen Molecular Biology, London School of Hygiene and Tropical Medicine, Keppel Street, London WC1E 7HT, UK; ^3^School of Biosciences, University of Exeter, Devon, UK

## Abstract

There is a requirement for an efficacious vaccine to protect people against infection from *Francisella tularensis*, the etiological agent of tularemia. The lipopolysaccharide (LPS) of *F. tularensis* is suboptimally protective against a parenteral lethal challenge in mice. To develop a more efficacious subunit vaccine, we have used a novel biosynthetic technique of protein glycan coupling technology (PGCT) that exploits bacterial N-linked glycosylation to recombinantly conjugate *F. tularensis* O-antigen glycans to the immunogenic carrier protein *Pseudomonas aeruginosa* exoprotein A (ExoA). Previously, we demonstrated that an ExoA glycoconjugate with two glycosylation sequons was capable of providing significant protection to mice against a challenge with a low-virulence strain of *F. tularensis*. Here, we have generated a more heavily glycosylated conjugate vaccine and evaluated its efficacy in a Fischer 344 rat model of tularemia. We demonstrate that this glycoconjugate vaccine protected rats against disease and the lethality of an inhalational challenge with *F. tularensis* Schu S4. Our data highlights the potential of this biosynthetic approach for the creation of next-generation tularemia subunit vaccines.

## 1. Introduction

Tularemia is caused by the intracellular bacterium *Francisella tularensis*. This bacterium can cause a range of presentations of disease in humans. In the most severe cases where infection is acquired by the pulmonary route, the mortality rate was found to be between 30 and 60% prior to the introduction of antibiotics [1]. The more virulent *F. tularensis* subsp. *tularensis* strains are endemic across North America. Lower virulence strains, including *F. tularensis* subsp. *holarctica* are endemic more widely in the Northern Hemisphere across Europe, America, and Asia. These high- and low-virulence strains are commonly designated as type A and type B strains, respectively [2]. Extrapolation of data from human aerosol infection studies has estimated that lung deposition of a single colony forming unit (CFU) may be sufficient to establish infection [3]. The bacterium is categorised by the US Centers for Disease Control and Prevention as a Tier 1 biological select agent due to its low infectious dose via the aerosol route and disease severity. Development of a safe and effective vaccine to protect against aerosol challenge with this bacterium remains a priority.

*F. tularensis* subsp. *holarctica* live vaccine strain (LVS) has been previously used in humans to protect against tularemia in at-risk populations such as laboratory workers. This vaccine was tested in humans experimentally and shown to protect against disease resulting from aerosol challenges of up to 20,000 CFU [4, 5]. Whilst demonstrating good efficacy, the mechanisms of its attenuation remain poorly defined. Phase II clinical trials to determine the safety and immunogenicity of LVS remain ongoing [6]. To provide a more defined alternative to LVS, several engineered live attenuated vaccines have been constructed which have demonstrated efficacy in animal models of disease [7–12]. In comparison with live attenuated candidates, safety compliance requirements for potential licensure are expected to be easier to achieve with subunit vaccines. However, overcoming efficacy limitations of subunit candidates has been the challenge to date. The only protein subunit candidate that has provided partial protection against type A strains of *F. tularensis* is IglC, but that was when delivery was through the use of a live attenuated *Listeria monocytogenes* vector [13]. Currently, lipopolysaccharide (LPS) is the only defined subunit *F. tularensis* vaccine antigen that has been reported to provide protection to immunised animals, although principally only against the lower virulence strains [14–17]. Therefore, whilst LPS remains a promising subunit candidate, strategies to improve its efficacy are warranted.

As LPS is a T cell-independent antigen, a strategy employed to enhance protective immunity for vaccines developed and licensed for other human pathogens is the incorporation of an antigenic carrier protein to the polysaccharide subunit. This approach has been successfully employed for several licensed public health vaccines including against *Neisseria meningitidis*, *Haemophilus influenzae* type B, and *Streptococcus pneumoniae* [18]. As proof of concept for the benefits of this approach in the field of tularemia, conjugation of *F. tularensis* LPS to bovine serum albumin induced protective immunity against type B, but not type A, strains of *F. tularensis* in mice [17].

These traditional conjugation approaches require the purification of the glycan from the native bacteria and then chemical conjugation of the glycans to a suitable carrier protein. This multistep approach can be time consuming, costly, and susceptible to variations between bioconjugation preparation batches. An alternative protein conjugation strategy adopted by our laboratory is the use of protein glycan coupling technology (PGCT) which facilitates the *in vitro* transfer of glycans to a recombinant acceptor protein using the glycosylating enzyme PglB from *Campylobacter jejuni* [19–22]. The presence of the PglB gene locus allows coupling of glucans to recombinantly expressed proteins containing the acceptor sequon D/E-X-N-Y-S/T, where X and Y are any amino acid except proline. We previously utilised PGCT to transfer recombinantly synthesized *F. tularensis* subsp. *tularensis* O-antigen to the carrier protein *Pseudomonas aeruginosa* exoprotein A (ExoA). This glycoconjugate was engineered to contain two glycosylation sequons and was produced using an *Escherichia coli* expression system [23]. We demonstrated that this glycoconjugate significantly improved the protection from disease in mice infected with *F. tularensis* subsp. *holarctica* compared to immunisation with LPS alone [23].

In the current study, we have introduced a further eight sequons into the sequence of ExoA resulting in a protein conjugate more highly glycosylated with *F. tularensis* O-antigen sugars. To allow stringent efficacy evaluation of this next-generation vaccine, we have developed a Fischer 344 (F344) rat inhalational challenge model and demonstrated that this subunit glycoconjugate vaccine can protect rats against an aerosol challenge of the high-virulence strain of *F. tularensis* Schu S4.

## 2. **Ma**t**erials and Methods**

### 2.1. *Francisella* Bacterial Strains and Culture

For vaccination of rats with LVS, a lyophilised vial of LVS (National Drug Biologic Research Company, USA, lot number 4) was reconstituted in phosphate-buffered saline (PBS, Life Technologies, UK), inoculated onto blood cysteine glucose agar (BCGA), and incubated at 37°C for 48 h. Bacterial growth was recovered from the agar and resuspended in PBS, and the optical density at 600 nm (OD_600_) was adjusted to 0.14. The suspension was serially diluted ten-fold to the desired concentration for immunisation.

For challenge studies, *F. tularensis* Schu S4 was inoculated onto BCGA and incubated at 37°C for 24 h. Growth was recovered from agar, resuspended in PBS, and the OD_600_ adjusted to 0.1. One mL of this suspension was inoculated into 100 mL of modified cysteine partial hydrolysate (MCPH) broth with 4% glucose and incubated with shaking at 180 rpm, at 37°C for 48 h. OD_600_ of the culture was adjusted to 0.1 in PBS and serially diluted to the desired concentration for aerosol challenge. Challenge inoculum quantification was determined by plating serially diluted cultures onto BCGA and incubating at 37°C for 48–72 h.

To determine bacterial load in organs, organs were weighed, homogenised through a 40 *μ*m cell sieve, serially diluted in PBS, plated onto BCGA, and incubated at 37°C for 48–72 h.

### 2.2. Production of the Glycoconjugate Vaccine (GtExoA)

#### 2.2.1. Bacterial Strains and Plasmid Construction

*Escherichia coli* CLM24 [24] was used as the host strain for protein expression and glycoconjugate production. CLM24 (a ligase negative strain) was stably transformed with the plasmid pGab2 [23], a construct created from the insertion of the *F. tularensis* subspecies *tularensis* strain Schu S4 O-antigen coding region into the low copy number expression plasmid pLAFR [25]. pGab2 is tetracycline selectable and constitutively expressed. Following confirmation of the expression of the *F. tularensis* O-antigen, the resulting strain was then transformed with the plasmid CLM24 containing a plasmid-encoded *C. jejuni* pglB, pGVXN114, which expresses the *C. jejuni* oligosaccharyltransferase PglB. Finally, the resulting strain was transformed with the plasmid pGVXN150: GtExoA, creating a three plasmid system for the production of the glycoconjugate. The GtExoA construct was engineered to express a modified version of *P. aeruginosa* exotoxin A that was synthesized by Celtek Bioscience LLC, USA in the vector pGH and closed into a vector derived from pEC415 using the restriction enzymes NheI and EcoRI (NEB, UK). The synthesized protein contains two internal modifications that allow glycosylation of the protein by PglB [23], as well as containing four *N-*glycosylation sequons at the N terminus and an additional 4 at the C terminus. In addition, a hexahistidine tag was added to the C terminus of the protein to facilitate purification and an *E. coli* DsbA signal peptide was added to the N-terminal sequences enabling Sec-dependent secretion to the periplasm. pGVXN150: GtExoA is ampicillin resistant and L-(+)-arabinose inducible. The construct sequence was then confirmed using Sanger sequencing with the primers GtExoA NF (GCGCTGGCTGGTTTAGTTT), GtExoA NR (CGCATTCGTTCCAGAGGT), GtExoA CF (GACAAGGAACAGGCGATCAG), and GtExoA CR (TGGTGATGATGGTGATGGTC).

#### 2.2.2. Culture and GtExoA Glycoprotein Expression Conditions

For all experiments, *E. coli* CLM24 was cultured in Luria-Bertani (LB) broth (Fisher Scientific, UK) supplemented with appropriate antibiotics in the following concentrations: ampicillin 100 *μ*g/mL, tetracycline 20 *μ*g/mL, and spectinomycin 80 *μ*g/mL. The addition of manganese chloride at the time of protein and PglB induction was at a final concentration of 4 mM, and made up as a 1 M stock fresh prior to each experiment. Cultures were incubated at 37°C shaking at 110 rpm for 16–20 hrs for large-scale preparation. For three-plasmid system glycoconjugate production, an overnight LB culture of *E. coli* CLM24 harbouring pGVXN114, pGVXN150: GtExoA, and pGab2 were subcultured in a 1 : 10 dilution of LB broth (Fisher Scientific) with antibiotics, and grown to mid log phase. pGVXN150: GtExoA was induced by the addition of 0.2% L-(+)-arabinose (Sigma-Aldrich, UK), and *C. jejuni* PglB was induced with 1 mM IPTG, followed by incubation for an initial 4 hours. Another addition of 0.4% L-(+)-arabinose was then added and cultures were incubated overnight.

#### 2.2.3. Production and Purification of Glycoconjugate Vaccine

1.8 L of LB was inoculated with a 200 mL starter culture and grown to an OD_590_ of 0.60–0.80, then induced as described above. The next day, induced glycoconjugate pellets were harvested via centrifugation at 5300 ×g at 4°C for 30 minutes and were resuspended in ice-cold lysis buffer (50 mM NaH_2_PO_4_, 300 mM NaCl, and 10 mM imidazole) containing 1 mg/mL lysozyme (Sigma-Aldrich) and 0.15 *μ*L Benzonase® Nuclease (Novagen®, UK). Lysis, wash, and elution buffer were all adjusted to pH 8 with 5 M NaOH. Resuspended cells were subjected to five rounds of lysis using a prechilled Stansted High Pressure Cell Disruptor (Stansted Fluid Power Ltd., UK) under 60,000 psi (410 MPa) in continuous mode. Cell debris was subsequently pelleted by spinning at 7840 ×g at 4°C for 60 minutes. The resulting supernatant was kept on ice whilst being loaded onto a GE Healthcare, UK, HIS-trap HP 1 mL column. Then, the column was washed in buffer containing 50.0 mM NaH_2_PO_4_, 300 mM NaCl, and 20 mM imidazole whilst attached to an AKTA purifier. Material was eluted and collected in 1 mL fractions with an imidazole gradient of 30–500 mM elution buffer that also contained 20% v/v glycerol and 5% w/v glucose. The collected fractions were visualised by Western blot, and the glycosylated GtExoA fractions were pooled and concentrated using buffer exchange columns (Vivaspin 2 (Vivaproducts, UK)) into PBS 20% v/v glycerol, prior to quantification with a BCA Protein Assay Kit (Pierce Biotechnology, USA).

#### 2.2.4. Western Blot Analysis

To assess protein expression and glycosylation levels, a two-channel simultaneous Western blot (Odyssey LI-COR, LI-COR Biosciences, Hamburg Germany) was used to analyse AKTA purified elution fractions. Freshly eluted samples were resuspended in 2x Laemmli buffer and boiled at 95°C for 6 minutes. Boilates, and a PageRuler Plus Prestained Protein Ladder (Life Technologies) were separated on a NuPAGE 10% Bis-Tris Gel Novex®, then transferred to a Hybond™-C Extra nitrocellulose membrane (Amersham Biosciences, UK). The membrane was then blocked in 2% w/v skim milk and PBS 0.2% v/v Tween 20 (Sigma-Aldrich) overnight at 4°C. The next day, membranes were probed simultaneously with two primary antibodies: O-antigen presence was detected using the mouse monoclonal antibody FB-11 (1 : 10,000) (Abcam, UK) and GtExoA was detected with rabbit anti-HIS polyclonal antibodies (1 : 5000) (Abcam). Secondary antibodies were Goat anti-Rabbit IRDye® 680RD and Goat anti-Mouse IRDye® 800CW (Odyssey® LI-COR Biosciences, UK) both diluted 1 : 10,000.

### 2.3. Animal Procedures

#### 2.3.1. Ethics Statement

Animals were kept in accordance with the UK Animals (Scientific Procedures) Act 1986 and Codes of Practice for the Housing and Care of Animals used in Scientific Procedures 1989. The license application underwent approval by the local ethical review process with the Defence Science and Technology Laboratory (Dstl) Animal Welfare and Ethical Review Body (AWERB) before submission and approval with the UK Home Office and Animal Procedures Committee (an independent committee that offers advice to The Secretary of State of the ethics of the proposed work). The project license that covered this work was 30/3166. No prespecified effect size was predicted for the glycoconjugate, and therefore no sample size estimate was made. No randomisation of animals or blinding of investigators was used in this study. No animals were excluded from the study.

#### 2.3.2. Animals

Female F344 rats were obtained from Envigo, UK. Rats were implanted with BioThermo microchips (Identipet, SA) by subcutaneous (s.c.) injection to allow individual rats to be tracked and have their temperature measured through the study. Rats used in vaccine studies were 12–16 weeks of age and weighed 190 ±20 g at the start of the procedures. On arrival in the conventional animal unit and on transfer of rats into containment level 3 animal facilities, rats were acclimatised to their new surroundings for 10 days before any procedures were performed. Rats were housed in cages of five, in polypropylene cages with a stainless steel mesh cover with an integral water bottle holder and diet hopper which conformed to the Code of Practice for the housing of animals bred, supplied, or used for scientific purposes (December 2014). Rats were kept under a 12 hour light/dark cycle (350 to 400 Lux during the day, 10 Lux during the night, with a ramp up and ramp down period at “dawn” and “dusk”) at 19 to 23°C and 45 to 65% relative humidity. Cages contained 8/10 and 10/14 grade corn cob (International Product Supplies, UK) as a nesting material with a range of environmental enrichment added throughout the studies (e.g., irradiated aspen wood, Des.Res. rat houses (LBS, UK)), and there was free access to food (Labdiet certified rodent diet 5002 and Labdiet EU rodent 22% diet 5LF5; International Product Supplies) and water throughout the study. During immunisation and the subsequent rest period, rats were housed in a conventional animal unit, in rooms supplied with rough filtered air giving 20 to 25 air changes per hour. For challenge with *F. tularensis* Schu S4, all animals were handled under UK Advisory Committee on Dangerous Pathogens animal containment level 3 conditions within a half-suit isolator compliant with British Standard BS5726, supplied with an inward flow of HEPA-filtered air giving 35 to 45 air changes per hour. The room was supplied with double HEPA-filtered air giving 20 to 25 air changes per hour.

#### 2.3.3. Experimental Animal Procedures

Rats were vaccinated with LVS in PBS via the s.c. route. Rats were vaccinated with 10 *μ*g GtExoA coadministered with the MF59 adjuvant (Novartis, UK) in a 100 *μ*L volume via the s.c. or intraperitoneal (i.p.) route 3 times, 2 weeks apart. Control groups of rats (*n* = 5) were also immunised by the i.p. and s.c. routes with the MF59 adjuvant alone or with PBS by the s.c. route. Aerosol challenge with *F. tularensis* Schu S4 occurred five weeks following final vaccination. Following challenge, animals were observed twice daily and signs of disease and subcutaneous temperature were recorded. Disease signs were assigned a score. The presence of piloerection and eye problems were scored given a clinical score of 1 or 2 depending on severity. Hunched posture, rapid breathing, and pinched posture were each given a clinical score of 1 if present. If any additional abnormal clinical signs were observed (e.g., pale tail), they were assigned a score of 1. A cumulative score for disease at each observed timepoint was calculated. Animals were weighed once daily. A humane endpoint was applied to rats in a moribund state or where their temperature was less than 33°C. Animals underwent euthanasia with intraperitoneally administered sodium pentobarbitone.

#### 2.3.4. Aerosol Challenge

Rats were exposed to an aerosol of *F. tularensis* Schu S4 by the inhalational route in a nose-only exposure unit (EMMS, UK) utilising a 6-jet Collison atomiser (Dstl, in-house) attached to a contained Henderson Piccolo arrangement to condition the aerosol to 50% (±5%) relative humidity. The nose-only exposure unit was controlled by the Aerosol Management Platform (AeroMP) aerosol system (Biaera Technologies L.L.C., USA). The animals were exposed to the aerosolised bacteria for 10 minutes, with impingement of the aerosol cloud sampled at the midway point of challenge into PBS via an All-Glass Impinger (AGI-30; Ace Glass, Vineland, NJ, USA). Following the challenge, the impinged aerosol was enumerated by serial dilution, plated onto BCGA plates, and incubated at 37°C for 48 h. The challenge dose was calculated from the aerosol concentration (CFU/L of air) using Guyton's formula [26] for minute respiratory volume and assuming 40% retention of 1–3 *μ*m droplets [27].

### 2.4. Immunological Assays

#### 2.4.1. Cell Isolation and Culture

Rat spleens were homogenised through a 40 *μ*m sieve using a sterile syringe plunger and collected into L15 medium (Life Technologies). The isolated splenocytes were diluted to 2 × 10^6^ cells/mL in medium and cultured in the presence of either medium alone, sonicated LVS whole cells (10 *μ*g/mL, Dstl), sonicated Schu S4 whole cells (10 *μ*g/mL, Dstl), purified ExoA (5 *μ*g/mL, London School of Hygiene and Tropical Medicine, UK), or Concanavalin-A (Con-A, 5 *μ*g/mL, Sigma-Aldrich). For cultures of cells from LVS-infected or PBS control rats, splenocytes were diluted in L15 medium supplemented with 10% foetal bovine serum (Sigma-Aldrich), nonessential amino acids (Life Technologies), 2-mercaptoethanol (Life Technologies), 100 U/mL penicillin, and 100 mg/mL streptomycin sulphate (Life Technologies) and then cultured at 37°C in the absence of a controlled CO_2_ environment. For cultures of cells from vaccinated rats, splenocytes were diluted in RPMI 1640 medium (Life Technologies), supplemented as described above, and then cultured at 37°C with 5% CO_2_.

#### 2.4.2. Measurement of IFN*γ* by Enzyme-Linked Immunosorbent Assay (ELISA)

Splenocytes (2 × 10^5^ per assay well) were cultured in duplicate in the presence of the antigen for 72 hours (see above), and supernatants were harvested and stored at −20°C prior to use. The expression of IFN*γ* was determined in plasma supernatants using a commercial rat IFN*γ* ELISA kit (Mabtech, Sweden) with responses determined by the measurement of optical density at 450 nm (OD_450nm_).

#### 2.4.3. ELISA for Anti-GtExoA Antibody Titre

Plates were coated with 5 *μ*g/mL GtExoA in PBS, 100 *μ*L per well, and incubated at 4°C overnight. After blocking with 1% skimmed milk powder in PBS for 2 hours at 37°C, plates were washed three times with 0.05% Tween 20 (Sigma-Aldrich) in PBS. Sera from individual rats were applied to plates at 1 : 50 and serially diluted 1 : 2 across the plate, in 1% skimmed milk powder. Bound IgG rat antibody was detected using anti-rat antibody conjugated to HRP at 1 : 2000 in PBS and developed using 10 mM 2,2′-azino-bis(3-ethylbenzothiazoline-6-sulphonic acid) in citrate buffer with 0.01% H_2_O_2_. OD was measured at 450 nm. Antibody titre was defined as the reciprocal of the highest dilution of serum that had a mean OD value at least 3 standard deviations higher than the mean OD of nonvaccinated serum.

### 2.5. Statistical Analysis

Analysis of rat weight data was performed using IBM SPSS version 21.0. All other statistical analyses were performed using GraphPad Prism version 6.02. The statistical tests applied to the different data sets are described in the corresponding figure legends.

## 3. Results

### 3.1. Production of the GtExoA Glycoconjugate Vaccine

**Figure 1:**
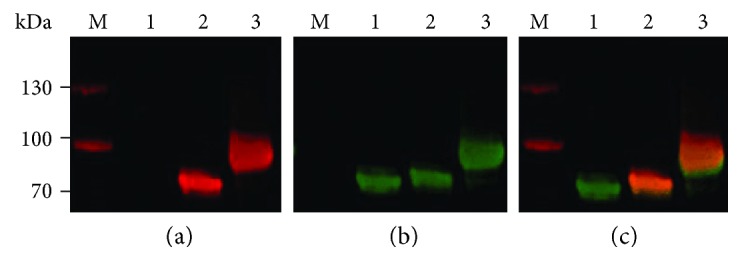
Recombinant ExoA modified to incorporate additional glycosylation sequons is heavily glycosylated by *F. tularensis* O-antigen by *C. jejuni* PglB in *E. coli* CLM24. Samples were separated by SDS-PAGE, and then two-colour Western blots were used to simultaneously detect the degree of glycosylation of ExoA using (a) a monoclonal mouse antibody (FB-11) with specificity to *F. tularensis* O-antigen (red) and (b) rabbit polyclonal antibodies with specificity to the 6x His sequence (green). The two IR secondary antibody channels when overlaid (IR 800/680) result in images with overall yellow colour indicating conjugation (c). M, protein ladder marker; lane 1, pGVXN150 only; lane 2, pGVXN150 ExoA glycosylated with the *F. tularensis* O-antigen (same construct from Cuccui et al. [23]); and lane 3, GtExoA heavily glycosylated with the *F. tularensis* O-antigen due to the presence of an additional eight glycosylation sequons.

The glycoconjugate vaccine previously evaluated by our group was glycosylated via two sequons incorporated into the ExoA carrier protein [23]. To improve the ratio of glycan to protein in the conjugate, a further 8 sequons were introduced into ExoA resulting in a more highly glycosylated conjugate, GtExoA. Recognition of GtExoA by a monoclonal antibody, FB-11, with specificity towards *F. tularensis* O-antigen demonstrated conservation of sugar moieties (Figure 1). No binding of FB-11 was observed in ExoA lacking the glycosylation sequons (Figure 1, lane 1). Western blot analysis of purified glycoconjugate vaccines demonstrated an increase in the molecular size of the decaglycosylated GtExoA compared with the biglycosylated first generation conjugate (Figure 1). This observation was commensurate with an expected increase in glycosylation resulting from the inclusion of the additional sequons. Due to the increased antigenic potential of this glycoconjugate vaccine, GtExoA was prioritised for efficacy evaluation.

### 3.2. Development of a F344 Rat Rodent Model of Inhalational Tularemia to Allow Efficacy Evaluation of Candidate Vaccines

**Figure 2:**
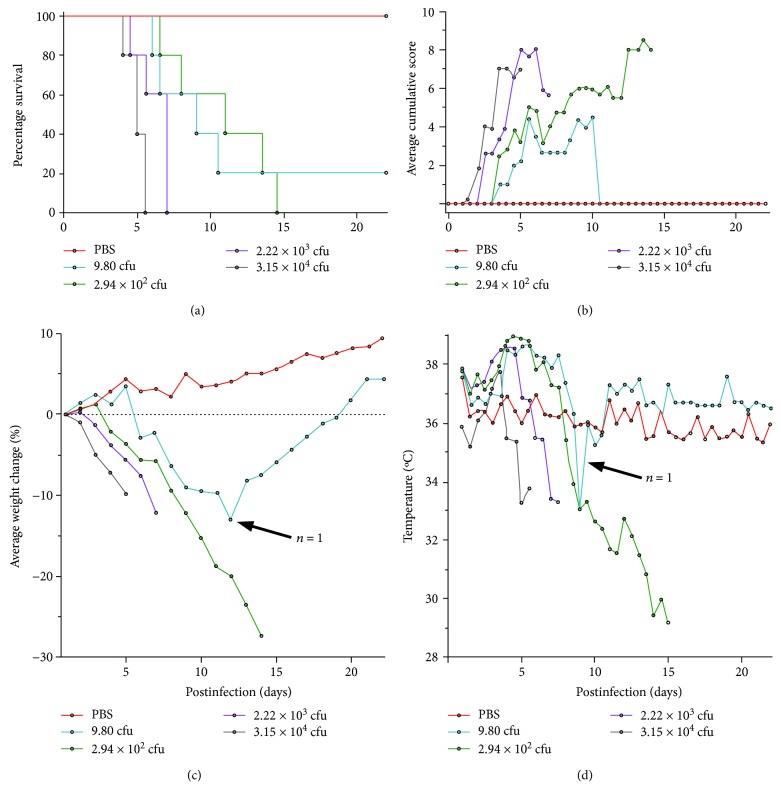
Survival and disease progression of rats following an aerosol challenge with a range of doses of *F. tularensis* Schu S4. (a) F344 rats (*n* = 5) were challenged via the aerosol route with a range of *F. tularensis* Schu S4 doses (see accompanying legend). Rats were monitored daily for mortality, and data were reported on the Kaplan-Meier survival curve. Calculated retained dose for each challenge group is shown on the Kaplan-Meier survival curve. (b) Clinical signs of disease were monitored twice daily. Average cumulative signs for each group are presented for animals which had not succumbed to disease. (c) Weight was monitored daily. Average weight change for each group is presented for animals which had not succumbed to disease. (d) Temperature was monitored twice daily. Average animal temperature for each group is presented for animals which had not succumbed to disease.

F344 rats have recently gained popularity as a preferred rodent model for assessing tularemia vaccines. In comparison with mice, F344 rats are considered to provide a closer approximation of human disease [28, 29] and demonstrate a more comparable response to LVS vaccination [30]. We therefore first developed an in-house F344 model of inhalational tularemia to allow stringent evaluation of GtExoA. Groups of 5 rats were challenged with a range of doses of *F. tularensis* Schu S4 via the aerosol route to determine an appropriate infectious dose. The estimated inhaled dose ranged from approximately 10 CFU to 3.15 × 10^4^ CFU. All rats challenged with 2.94 × 10^2^ to 3.15 × 10^4^ CFU succumbed to infection within 14 days of challenge (Figure 2(a)). Of rats challenged with approximately 10 CFU, only 1 animal out of 5 survived to the end of the experiment at 21 days postinfection. During the recovery of this animal, its disease signs resolved and some weight was recovered. The mean lethal dose (MLD) was therefore estimated to be less than 10 CFU via the aerosol route in our model.

Bacterial dissemination was determined at day 7 postchallenge in groups of up to five sacrificed rats. Animals sacrificed at this time had highly colonised lungs, liver, and spleens (Supplementary Figure S1). All infected rats showed severe signs of disease. Rats initially exhibited piloerection and developed eye problems, including secretion of porphyrin and ptosis of the eyelids until their eyes were completely closed, followed by a hunched posture alongside rapid breathing. Rats became more lethargic and less responsive to stimuli over the course of disease (Figure 2(b)). All infected rats lost weight in a dose-dependent manner (Figure 2(c)) and displayed a febrile stage, with subcutaneous temperatures which were raised at least 1.5°C above their baseline temperature (Figure 2(d)). Rats which received the highest challenge of 3.15 × 10^4^ CFU rapidly lost between 7 and 10 percent of their body weight within 5 days of challenge. Those rats which received a lower challenge all lost at least 10 percent of their starting weight, and in some cases more than 25 percent of body weight, but over a greater length of time. These data were used to identify disease parameters useful for assessing candidate vaccine performance in the model.

### 3.3. GtExoA Glycoconjugate Vaccine Induces Memory Immunity in Vaccinated F344 Rats

**Figure 3:**
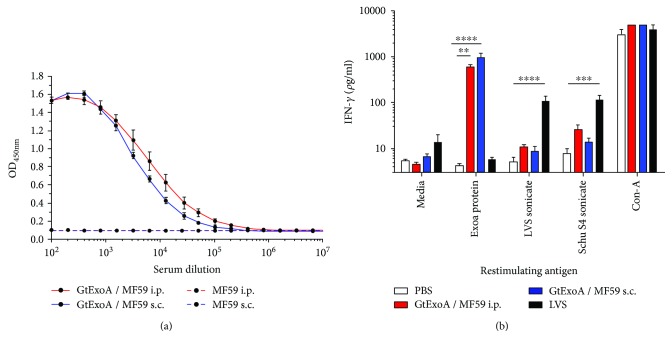
GtExoA glycoconjugate stimulates memory immunity in vaccinated rats. Groups of F344 rats (*n* = 5) were vaccinated three times, two weeks apart with 10 *μ*g of GtExoA coadministered with MF59, or immunised with MF59 alone, and immune responses were measured 28 days after the third immunisation. (a) Quantitation of rat IgG recognising the GtExoA glycoconjugate antigen was determined by ELISA for a serial dilution of sera from GtExoA and respective MF59 adjuvant control rats. The mean OD_450nm_ (±SEM) response is presented for each dilution for each vaccine group. The use of solid or dotted datapoint connecting lines identifies responses in sera from rats immunised with GtExoA + MF59 or with the MF59 adjuvant only, respectively. Responses in respective groups immunised via the i.p. or s.c. routes are identified using red or blue connecting lines, respectively (see legend). (b) Splenocytes were isolated from rats immunised with either GtExoA administered by i.p. (light blue bars) or s.c. (dark blue bars) routes and from rats immunised with LVS (black bars) or PBS. Splenocytes were cultured in the presence of purified ExoA protein, LVS sonicate, *F. tularensis* Schu S4 sonicate, Con-A, or medium. The expression of IFN*γ* in 72-hour culture supernatants was measured by ELISA. The OD_450nm_ results were normalised by transformation into units of *ρ*g/mL by generating a standard curve using recombinant rat IFN*γ*. Statistical analysis of differences between groups was determined by one-way ANOVA with Holm-Sidak's posttests (^∗∗^ *p* < 0.01, ^∗∗∗^ *p* < 0.001, or ^∗∗∗∗^ *p* < 0.0001). Data validity was tested using Bartlett's test for equal variance. IFN*γ* responses are presented as mean response for each group (*n* = 5) ±SEM.

To determine whether the GtExoA glycoconjugate vaccine could induce memory immunity in rats prior to proceeding to a biosafety level 3 efficacy challenge study, groups of 5 rats were vaccinated with GtExoA in combination with the MF59 adjuvant. Groups were vaccinated by i.p. or s.c. administration routes. We previously used the i.p. vaccination route to assess the first generation glycoconjugate vaccine in mice [13], whilst s.c. is the immunisation route routinely used for LVS, the tularemia gold-standard reference vaccine. Therefore, both immunisation routes were assessed to allow translation between mouse and rat models and to control for the s.c. immunisation route used for LVS administration. Rats were vaccinated on 3 occasions, 2 weeks apart. Control groups included PBS sham-vaccinated rats, MF59 adjuvant-only-immunised rats and a group vaccinated with LVS. Serum and splenocytes were recovered 28 days after the final vaccination to measure IgG- and cell-mediated responses, respectively. Sera from the MF59 adjuvant-only-vaccinated controls showed no appreciable binding to the GtExoA antigen, whilst endpoint IgG titres from rats vaccinated with GtExoA by the i.p. or s.c. routes were 1 : 204800 and 1 : 102400, respectively (Figure 3(a)). Antigen-stimulated expression of IFN*γ* was used as a measure of T cell-mediated memory. Significantly elevated ExoA-stimulated IFN*γ* responses were only observed in rats immunised with GtExoA (Figure 3(b)) confirming the recognition of the ExoA conjugate protein by the cell-mediated compartment of these rats. No increase in ExoA-stimulated IFN*γ* from splenocytes isolated from rats immunised with the MF59 adjuvant by i.p. or s.c. routes was observed (data not shown). Stimulation of splenocytes with the crude *F. tularensis* antigen preparations only resulted in significantly elevated IFN*γ* expression in the group of rats vaccinated with LVS (Figure 3(b)). Elevated IFN*γ* responses stimulated by *F. tularensis* Schu S4 sonicate in the GtExoA-vaccinated groups of rats, potentially as a consequence of nonspecific stimulation by components in this crude antigen extract, were not significantly stronger than responses observed in PBS-immunised rats (Figure 3(b)) or control rats immunised with MF59 alone (data not shown).

### 3.4. GtExoA Glycoconjugate Protects against Pulmonary Tularemia in F344 Rats

**Figure 4:**
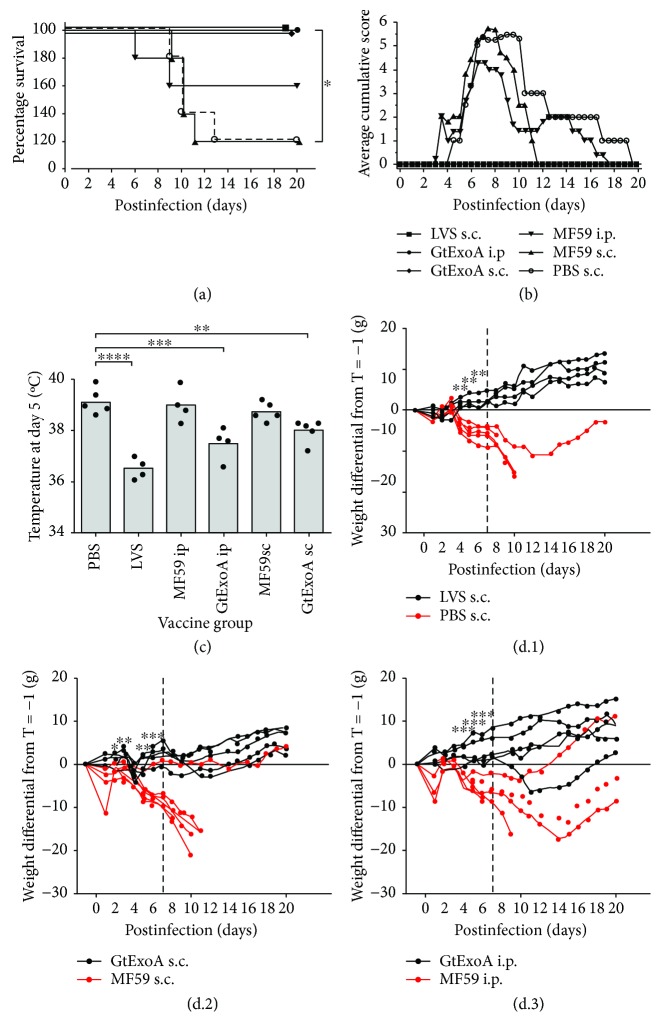
GtExoA protects F344 rats against aerosol infection with *F. tularensis* Schu S4. Groups of F344 rats (*n* = 5) were vaccinated three times, two weeks apart with 10 *μ*g GtExoA coadministered with MF59, or immunised with MF59 alone, via the s.c. or i.p. route, or 5.38 × 10^7^ LVS. 5 weeks after final vaccination, rats were challenged with a calculated retained dose of 5.48 × 10^2^ *F. tularensis* Schu S4 via the aerosol route. (a) Rats were monitored twice daily and mortality and survival plotted on a Kaplan-Meier survival curve. For comparison of survival curves, a log-rank Mantel-Cox test was used (^∗^ *p* < 0.05). (b) Signs of disease were recorded twice daily and average cumulative signs for surviving rats in each group of 5 presented. (c) The temperature for individual rats (black circles) in each treatment group is presented at day 5 postinfection, the day on which the maximal febrile temperature was detected. The grey bar is the average response for the group. Statistical analysis of differences between the temperatures of treatment and the PBS control group was determined by one-way ANOVA with Holm-Sidak's posttests (^∗∗^ *p* < 0.01, ^∗∗∗^ *p* < 0.001, ^∗∗∗∗^ *p* < 0001). (d.1–d.3) Rats were weighed once daily and average weight change of surviving rats is presented. Each group is presented with its apposite control: LVS s.c. (black lines) and PBS s.c. (red lines) (d.1), GtExoA + MF59 s.c. (black lines) and MF59 s.c. (red lines) (d.2), and GtExoA + MF59 i.p. (black lines) and MF59 i.p. (red lines) (d.3). Significance in divergence of weight change between groups was found to fit the normal, Gaussian distribution using Q-Q plots (not shown). The data was analysed using a repeated measures general linear model. Validity of the data for this test was further established using Levene's tests for unequal variance (not shown). Individual comparisons, pairwise and dependent or independent of timepoints, were performed using Bonferroni's correction (^∗^ *p* < 0.05, ^∗∗^ *p* < 0.005, and ^∗∗∗^ *p* < 0.0005). Due to culling of rats that reached their humane endpoint, the limit of analysis depicted by the dotted line is the timepoint up to which statistical comparisons could be performed across all groups with equivalent power.

To evaluate the efficacy of our glycoconjugate vaccine, groups of 5 rats were vaccinated with the GtExoA by both the s.c. and i.p. routes along with appropriate MF59 adjuvant controls. A group of rats (*n* = 5) was also vaccinated with 5 × 10^7^ CFU LVS via the s.c. route. The LVS group was included to validate the relevance of the model, whilst also providing a reference gold standard against which to assess the performance of our GtExoA vaccine candidate. Five weeks after the final vaccination, rats were challenged with an aerosol of 5.48 × 10^2^ *F. tularensis* Schu S4 (the calculated retained dose). All LVS- and GtExoA bioconjugate-vaccinated rats survived 21 days postaerosol challenge (Figure 4(a)). One of the five rats vaccinated with the MF59 adjuvant alone via the s.c. route survived to 21 days postinfection but the group survival curve was still significantly different from rats vaccinated via the s.c. route with GtExoA (*p* < 0.05) (Figure 4(a)). Similarly, despite one of the five PBS-immunised rats surviving 21 days postinfection, survival was significantly different from rats vaccinated via the s.c. route with LVS (Figure 4(a), *p* < 0.05). However, as a consequence of three of the five rats vaccinated with MF59 alone via the i.p. route not succumbing to a lethal infection, the difference between their survival and that of the comparable i.p. GtExoA vaccine group did not reach significance (*p* = 0.168, log-rank test). The level of significance for the comparison of all survival curves is presented in Supplementary Table S1.

Rats vaccinated with GtExoA by either route, or with LVS, showed no clinical signs of disease (Figure 4(b)) and did not become febrile (Supplementary Figure S2). In contrast, rats vaccinated with PBS subcutaneously and MF59 only via i.p. and s.c. routes all became febrile with maximal febrile temperature being observed on day 5 postinfection. On day 5, the temperature of rats in the groups vaccinated with LVS or GtExoA (i.p. and s.c. routes) were all significantly lower than those in the PBS control group (*p* < 0.001, 0.001, and 0.01, respectively, ANOVA with Dunnett's postanalysis test), see Figure 4(c). There was no significant difference between the temperature of the PBS or MF59 (i.p. and s.c. routes) adjuvant control rats. Furthermore, all PBS- and MF59-only-immunised rats showed clinical signs of disease including those that did not ultimately succumb to a lethal infection (Figure 4(b)).

Weight change in rats after challenge was shown to be significantly different over time between all groups of vaccinated rats and their relevant controls (Figure 4(d)). Weight change in rats vaccinated with LVS or with GtExoA via the i.p. route significantly diverged from their relevant controls on day 4 (Figures 4(d.1) and 4(d.3), *p* < 0.005, *p* < 0.0005, respectively). Weight change between rats vaccinated with GtExoA by the s.c. route and the relevant control rats significantly diverged on day 2 after challenge (Figure 4(d.2), *p* < 0.05). The control rats that did not reach a humane endpoint all resolved signs of disease and had recovered some weight by 21 days following infection. *F. tularensis* was not detected in the lungs, liver, or spleen of LVS- or GtExoA-vaccinated rats at 21 days postinfection whilst all surviving MF59- or PBS-only-vaccinated rats were colonised with *F. tularensis* in the lung, liver, and spleen at 21 days postinfection.

## 4. Discussion

The development of a subunit vaccine that can protect against inhalational infection with type A strains of *F. tularensis* remains an important goal for tularemia vaccine research. To this end, we have utilised PGCT to recombinantly express the immunogenic *P. aeruginosa* carrier protein ExoA glycosylated with O-antigen sugars of *F. tularensis* in a single-step process. We previously used this approach to engineer a glyconjugate incorporating two sequons and demonstrated the protective potential of this biglycosylated vaccine in a murine model of tularemia [23]. Here, we have engineered a second generation vaccine by introducing a further 8 sequons into the conjugate protein ExoA to increase the antigen potential of the glycoconjugate. The glycans were recognised by a monoclonal antibody with specificity for the terminal moiety 4,6-dideoxy-4-formamido-D-glucose of the *F. tularensis* subsp. *tularensis* and subsp. *holarctica* O-antigen [31]. The recognition by this antibody confirmed the presence of structurally conserved sugars. Furthermore, increasing the number of sequons in the second generation conjugate successfully resulted in a more heavily glycosylated conjugate, as confirmed by its increased molecular size. Together, these data demonstrate the versatility of this technology for generating glycoconjugate vaccine candidates. It is currently unclear how many repeating units are transferred by *C. jejuni* PglB in this system. The native *F. tularensis* O-antigen consists of a repeating tetrasaccharide structure [14]. The absence of a ladder of multiple-sized products separated by SDS-PAGE suggests that GtExoA principally carries single repeat units at its sequon sites. Efforts to modulate the level of *C. jejuni* PglB to increase the chain length of repeat glycan units is ongoing in the Wren laboratory.

Recent development of a F344 rat model of respiratory tularemia for testing vaccine candidates has provided the opportunity for testing candidates in a closer approximation of human disease [28, 29]. Tularemia in rats is less acute than in mice, reflecting human disease progression more closely. In addition, F344 rats show similar sensitivity to *F. tularensis* strains as humans [32] and rats can be protected from disease by vaccination with LVS [30]. We consider that establishing an aerosol-initiated rat model of tularemia at our centre, commensurate with that developed by Hutt et al. [29], to be an important step in allowing efficacy evaluation of both GtExoA and future subunit tularemia vaccines. In our aerosol challenge model, lethal infection could be established with less than 10 CFU of *F. tularensis* Schu S4. This confirms the disease susceptibility of F344 rats to inhalation of type A strains of *F. tularensis* reported previously [28, 29, 33]. LVS has been shown to protect F344 rats against respiratory infection by *F. tularensis* Schu S4 delivered by the aerosol route [34] and more recently by the intratracheal route [28]. The inclusion of LVS as a comparative reference vaccine in our efficacy study confirms that this is also the case following infection by the aerosol delivery methodology employed in our study. These data mimic protection invoked by LVS against *F. tularensis* delivered by the aerosol route in humans reported during human experiments in the 1960s [5, 35], supporting the value of this model for efficacy evaluation of tularemia vaccine candidates.

Analysis of the GtExoA Kaplan-Meier survival curves indicated that a significant survival benefit was observed for the s.c. but not i.p. immunisation route when compared with the corresponding MF59 adjuvant control rats. This was due to survival of 3/5 rats that received the MF59 adjuvant by the i.p. route. Although we allowed a 5-week interval between the final vaccination and challenge with *F. tularensis*, we would hypothesise that i.p. immunisation with MFP59 results in a prolonged stimulation of innate immunity. However, it should be noted that none of the GtExoA-vaccinated rats, regardless of immunisation route, showed clinical signs of disease or demonstrated weight loss. In contrast, all of the control rats, including all those that did not succumb to the lethal infection, showed weight loss and adverse clinical signs. Therefore, even where significant protection against lethality was not observed, complete protection against clinical disease was. Given that pneumonic infections by high-virulence strains of *F. tularensis* exhibited <60% mortality in humans in the preantibiotic era [1], we would advocate the benefits of the rat model in being able to measure protection against lethal and nonlethal disease outcomes. The inclusion of the i.p. immunisation route in this study was primarily to provide consistency with the route used in our previous mouse efficacy study [19]. We would not envisage this as an appropriate immunisation route for future clinical extrapolation.

The protection provided by GtExoA delivered by the s.c. route was comparable to LVS in this study. This is a significant achievement for a tularemia subunit vaccine in view of the use of an aerosol challenge model using a type A strain of *F. tularensis*. Whilst undoubtedly promising, it would be premature to state the vaccine to be as protective as LVS. Since the data presented is derived from a single efficacy study, it provides proof of concept of efficacy in this preliminary study. The challenge dose employed in this study was approximately 500 CFU. LVS has demonstrated that it can protect F344 rats against lethal challenges approaching 10^5^ CFU of *F. tularensis* Schu S4 delivered by the i.t. route [28]. Therefore, it will be important to test the efficacy of GtExoA in future dose escalation challenge studies to fully establish its protective potential compared with the efficacy bench mark set by LVS.

Since the O-antigen of *F. tularensis* is a T cell-independent antigen, we would hypothesise that the protection observed was principally mediated by the generation of protective antibody responses. We detected strong titres of glycoconjugate-specific IgG in serum from GtExoA-vaccinated rats. Whilst O-antigen is widely acknowledged as a serodominant antigen, we did not formally quantitate the relative contribution of the O-antigen and ExoA protein-specific IgG. In mice, we previously demonstrated that the conjugation of the O-antigen glycans to the carrier protein ExoA resulted in enhanced antibody concentrations compared with using LPS alone [13]. This was believed to be due to ExoA providing T cell help to promote more efficient antibody generation. In the current study, we confirmed that vaccination of rats with GtExoA also resulted in the generation of ExoA-specific cell-mediated immunity supporting this hypothesis. Understanding the immunological basis and duration of the protective immunity generated by our glycoconjugate vaccine will be an important consideration for future studies.

The next step toward the development of glycoconjugate vaccines produced by this PGCT technology would be to incorporate *F. tularensis* peptide antigens as the O-antigen carrier protein, rather than *P. aeruginosa* ExoA. This approach has been successfully applied to a PGCT-produced *Staphylococcus aureus* glycoconjugate vaccine. Switching the carrier protein from ExoA to the *S. aureus*-specific protein Hla resulted in improved vaccine efficacy [36]. Whilst no single peptide antigen has been shown to be protective as a vaccine candidate for *F. tularensis* type A strains in mice, several recombinant *F. tularensis* proteins have been shown to invoke a cellular immune response [37–39]. Moreover, encapsulated recombinant peptide antigens [40], and recently a multiantigen Tobacco Mosaic virus-based vaccine [41], have been shown to protect against lethal LVS challenge to mice. An *F. tularensis* antigen expressed as part of a whole cell vaccine platform has also been shown to boost efficacy of a live attenuated vaccine [13]. It is therefore considered that incorporation of immunogenic *F. tularensis* antigens into a glycoconjugate vaccine is a desirable next step in the development of this candidate. A combination of the humoral response to the O-antigen, boosted by T cell help due to conjugation to protein, alongside the cellular response to *F. tularensis-*specific T cell epitopes has the potential to improve the protection demonstrated by the existing candidate. Future optimisation of dosing schedules and choice of adjuvant will also be important development considerations.

## 5. Conclusions

We have utilised PGCT technology to produce an *F. tularensis* O-antigen ExoA glycoconjugate vaccine. We have developed a F344 rat aerosol challenge model which has been used to generate proof of concept data demonstrating that this O-antigen glycoconjugate vaccine can protect against an aerosol challenge of *F. tularensis* Schu S4. Testing of the next generation of glycoconjugate vaccine candidates in this rat model of aerosol-delivered *F. tularensis* should allow delineation of the efficacies of this new source of candidates and would be the next strategic step towards development of a protective and licensable human vaccine to protect against tularemia.

## Data Availability

The data used to support the findings of this study are available from the corresponding author upon request.

## Supplementary Materials

Supplementary Materials Supplementary materials contain two data figures and a table supporting the analysis, interpretation, and discussion of associated study results. Figure S1: bacterial burden of *F. tularensis* Schu S4 in tissues of F344 rats 7 days after aerosol infection. Figure S2: temperature profile of vaccinated F344 rats following *F. tularensis* Schu S4 aerosol infection. Table S1: statistical comparison of Kaplan-Meier survival curves in groups of vaccinated F344 rats (*n* = 5) challenged with *F. tularensis* Schu S4 by the aerosol route.

## References

[1] Stuart B. M., Pullen R. L. (1945). Tularemic pneumonia—review of American literature and reports of 15 additional cases. *American Journal of the Medical Sciences*.

[2] Jellison W. (1974). *Tularemia in North America. 1930-1974*.

[3] Jones R. M., Nicas M., Hubbard A., Sylvester M. D., Reingold A. (2005). The infectious dose of *Francisella tularensis* (tularemia). *Applied Biosafety*.

[4] Hornick R. B., Eigelsbach H. T. (1966). Aerogenic immunization of man with live tularemia vaccine. *Bacteriological Reviews*.

[5] Saslaw S., Wilson H. E., Prior J. A., Carhart S. R. (1959). Studies on the evaluation of tularemia vaccines in man. *Journal of Laboratory and Clinical Medicine*.

[6] Cardile A. Continued safety and immunogenicity study of a live *Francisella tularensis* vaccine. https://clinicaltrials.gov2016.

[7] Ireland P. M., LeButt H., Thomas R. M., Oyston P. C. F. (2011). A *Francisella tularensis* SCHU S4 mutant deficient in (gamma)-glutamyltransferase activity induces protective immunity: characterization of an attenuated vaccine candidate. *Microbiology*.

[8] Golovliov I., Twine S. M., Shen H., Sjostedt A., Conlan W. (2013). A ΔclpB mutant of *Francisella tularensis* subspecies *holarctica* strain, FSC200, is a more effective live vaccine than *F. tularensis* LVS in a mouse respiratory challenge model of tularemia. *PLoS One*.

[9] Twine S., Shen H., Harris G. (2012). BALB/c mice, but not C57BL/6 mice immunized with a ΔclpB mutant of *Francisella tularensis* subspecies *tularensis* are protected against respiratory challenge with wild-type bacteria: association of protection with post-vaccination and post-challenge immune responses. *Vaccine*.

[10] Mahawar M., Rabadi S. M., Banik S. (2013). Identification of a live attenuated vaccine candidate for tularemia prophylaxis. *PLoS One*.

[11] Signarovitz A. L., Ray H. J., Yu J. J. (2012). Mucosal immunization with live attenuated *Francisella novicida* U112ΔiglB protects against pulmonary *F. tularensis* SCHU S4 in the Fischer 344 rat model. *PLoS One*.

[12] Chu P., Cunningham A. L., Yu J.-J. (2014). Live attenuated *Francisella novicida* vaccine protects against *Francisella tularensis* pulmonary challenge in rats and non-human primates. *PLoS Pathogens*.

[13] Jia Q., Lee B.-Y., Clemens D. L., Bowen R. A., Horwitz M. A. (2009). Recombinant attenuated *Listeria monocytogenes* vaccine expressing *Francisella tularensis* IglC induces protection in mice against aerosolized type A *F. tularensis*. *Vaccine*.

[14] Prior J. L., Prior R. G., Hitchen P. G. (2003). Characterization of the O antigen gene cluster and structural analysis of the O antigen of *Francisella tularensis* subsp. *tularensis*. *Journal of Medical Microbiology*.

[15] Fulop M., Mastroeni P., Green M., Titball R. W. (2001). Role of antibody to lipopolysaccharide in protection against low- and high-virulence strains of *Francisella tularensis*. *Vaccine*.

[16] Fulop M., Manchee R., Titball R. (1995). Role of lipopolysaccharide and a major outer membrane protein from *Francisella tularensis* in the induction of immunity against tularemia. *Vaccine*.

[17] Conlan J. W., Shen H., Webb A., Perry M. B. (2002). Mice vaccinated with the O-antigen of *Francisella tularensis* LVS lipopolysaccharide conjugated to bovine serum albumin develop varying degrees of protective immunity against systemic or aerosol challenge with virulent type A and type B strains of the pathogen. *Vaccine*.

[18] Berti F., Adamo R. (2013). Recent mechanistic insights on glycoconjugate vaccines and future perspectives. *ACS Chemical Biology*.

[19] Szymanski C. M., Yao R., Ewing C. P., Trust T. J., Guerry P. (1999). Evidence for a system of general protein glycosylation in *Campylobacter jejuni*. *Molecular Microbiology*.

[20] Terra V. S., Mills D. C., Yates L. E., Abouelhadid S., Cuccui J., Wren B. W. (2012). Recent developments in bacterial protein glycan coupling technology and glycoconjugate vaccine design. *Journal of Medical Microbiology*.

[21] Wacker M., Linton D., Hitchen P. G. (2002). N-linked glycosylation in *Campylobacter jejuni* and its functional transfer into *E. coli*. *Science*.

[22] Wacker M., Feldman M. F., Callewaert N. (2006). Substrate specificity of bacterial oligosaccharyltransferase suggests a common transfer mechanism for the bacterial and eukaryotic systems. *Proceedings of the National Academy of Sciences of the United States of America*.

[23] Cuccui J., Thomas R. M., Moule M. G. (2013). Exploitation of bacterial N-linked glycosylation to develop a novel recombinant glycoconjugate vaccine against *Francisella tularensis*. *Open Biology*.

[24] Feldman M. F., Wacker M., Hernandez M. (2005). Engineering N-linked protein glycosylation with diverse O antigen lipopolysaccharide structures in *Escherichia coli*. *Proceedings of the National Academy of Sciences of the United States of America*.

[25] Karlsson J., Prior R. G., Williams K. (2000). Sequencing of the *Francisella tularensis* strain Schu 4 genome reveals the shikimate and purine metabolic pathways, targets for the construction of a rationally attenuated auxotrophic vaccine. *Microbial and Comparative Genomics*.

[26] Guyton A. C. (1947). Measurement of the respiratory volumes of laboratory animals. *American Journal of Physiology-Legacy Content*.

[27] Harper G. J., Morton J. D. (1953). The respiratory retention of bacterial aerosols—experiments with radioactive spores. *Journal of Hygiene*.

[28] Wu T. H., Zsemlye J. L., Statom G. L. (2009). Vaccination of Fischer 344 rats against pulmonary infections by *Francisella tularensis* type A strains. *Vaccine*.

[29] Hutt J. A., Lovchik J. A., Dekonenko A., Hahn A. C., Wu T. H. (2017). The natural history of pneumonic tularemia in female Fischer 344 rats after inhalational exposure to aerosolized *Francisella tularensis* subspecies *tularensis* strain SCHU S4. *American Journal of Pathology*.

[30] Mara-Koosham G., Hutt J. A., Lyons C. R., Wu T. H. (2011). Antibodies contribute to effective vaccination against respiratory infection by type A *Francisella tularensis* strains. *Infection and Immunity*.

[31] Roche M. I., Lu Z., Hui J. H., Sharon J. (2011). Characterization of monoclonal antibodies to terminal and internal O-antigen epitopes of *Francisella tularensis* lipopolysaccharide. *Hybridoma*.

[32] Ray H. J., Chu P., Wu T. H. (2010). The Fischer 344 rat reflects human susceptibility to *Francisella* pulmonary challenge and provides a new platform for virulence and protection studies. *PLoS One*.

[33] Raymond C. R., Conlan J. W. (2009). Differential susceptibility of Sprague-Dawley and Fischer 344 rats to infection by *Francisella tularensis*. *Microbial Pathogenesis*.

[34] Jemski J. V. (1981). Respiratory tularemia: comparison of selected routes of vaccination in Fischer 344 rats. *Infection and Immunity*.

[35] Saslaw S., Wilson H. E., Carhart S., HT E., Prior J. A. (1961). Tularemia vaccine studies. 2. Respiratory challenge. *Archives of Internal Medicine*.

[36] Wacker M., Wang L., Kowarik M. (2014). Prevention of *Staphylococcus aureus* infections by glycoprotein vaccines synthesized in *Escherichia coli*. *The Journal of Infectious Diseases*.

[37] Hartley M. G., Green M., Choules G. (2004). Protection afforded by heat shock protein 60 from *Francisella tularensis* is due to copurified lipopolysaccharide. *Infection and Immunity*.

[38] Golovliov I., Ericsson M., Akerblom L., Sandstrom G., Tarnvik A., Sjostedt A. (1995). Adjuvanticity of ISCOMs incorporating a T cell-reactive lipoprotein of the facultative intracellular pathogen *Francisella tularensis*. *Vaccine*.

[39] Sjostedt A., Sandstrom G., Tarnvik A. (1992). Humoral and cell mediated immunity in mice to a 17 kilodalton liprotein of *Francisella tularensis* expressed by *Salmonella typhimurium*. *Infection and Immunity*.

[40] Hickey A. J., Hazlett K. R. O., Kirimanjeswara G. S., Metzger D. W. (2011). Identification of *Francisella tularensis* outer membrane protein A (FopA) as a protective antigen for tularemia. *Vaccine*.

[41] McCormick A. A., Shakeel A., Yi C., Kaur H., Mansour A. M., Bakshi C. S. (2018). Intranasal administration of a two-dose adjuvanted multi-antigen TMV-subunit conjugate vaccine fully protects mice against *Francisella tularensis* LVS challenge. *PLoS One*.

