# Supplementary material for: An O-Antigen Glycoconjugate Vaccine Produced Using Protein Glycan Coupling Technology Is Protective in an Inhalational Rat Model of Tularemia
Source: J Immunol Res. 2018 Nov 29;2018:8087916. doi: 10.1155/2018/8087916 (PMC6304830; doi:10.1155/2018/8087916)
Supplement: Supplementary Materials — contain two data figures and a table supporting the analysis, interpretation, and discussion of associated study results. Figure S1: bacterial burden of F. tularensis Schu S4 in tissues of F344 rats 7 days after aerosol infection. Figure S2: temperature profile of vaccinated F344 rats following F. tularensis Schu S4 aerosol infection. Table S1: statistical comparison of Kaplan-Meier survival curves in groups of vaccinated F344 rats (n = 5) challenged with F. tularensis Schu S4 by the aerosol route. [file 8087916.f1.docx]

**Supplementary Figures and Table**

**

**

**Figure S1. Bacterial burden of *F. tularensis* Schu S4 in tissues of F344 rats 7 days after aerosol infection.** F344 rats (n=5) were challenged with a range of aerosol doses of *F. tularensis* Schu S4. Enumeration of bacteria was performed at day 7 post infection for all rats that survived to this time point. The bacterial burden (cfu/g) in homogenised lung, liver and spleen is presented for individual rats for each of the calculated retained challenge doses of 9.8 cfu (open circles), 2.9x10^2^ cfu (open squares) and 2.2x10^3^ cfu (open triangles).





**Figure S2. Temperature profile of vaccinated F344 rats following *F. tularensis* Schu S4 aerosol infection.** Groups of F344 rats (n=5) were vaccinated three times, two weeks apart with 10 µg GtExoA in MF59 or MF59 alone via the s.c. or i.p. route, or 5.38 x 10^7^ LVS. 5 weeks after final vaccination, rats were challenged with a calculated retained dose of 5.48 x 10^2^ *F. tularensis* Schu S4 via the aerosol route. Rats temperatures were recorded twice daily. Each group is presented with its apposite control: Panel A: LVS s.c. (black lines) and PBS s.c. (red lines). Panel B: GtExoA i.p. (black lines) and MF59 i.p. (red lines). Panel C: GT-ExoA s.c. (black lines) and MF59 i.p. (red lines).

**Supplementary Table S1.** Statistical comparison of Kaplan Meier survival curves in groups of vaccinated F344 rats (n=5) challenged with *F. tularensis* Schu S4 by the aerosol route. Comparison of curves by Log-rank (Mantel-Cox) test.

| Vaccine Group | Vaccine Group | | | | | |
| --- | --- | --- | --- | --- | --- | --- |
|  | 1 | 2 | 3 | 4 | 5 | 6 |
| 1. PBS s.c. | N/A | 0.9146 | 0.7463 | **0.0168** | **0.0168** | **0.0168** |
| 2. MF59 s.c. | 0.9146 | N/A | 0.7463 | **0.0168** | **0.0168** | **0.0168** |
| 3. MF59 i.p. | 0.7463 | 0.7463 | N/A | 0.1360 | 0.1360 | 0.1360 |
| 4. GtExoA + MF59 s.c. | **0.0168** | **0.0168** | 0.1360 | N/A | 1.000 | 1.000 |
| 5. GtExoA + MF59 i.p. | **0.0168** | **0.0168** | 0.1360 | 1.000 | N/A | 1.000 |
| 6. LVS s.c. | **0.0168** | **0.0168** | 0.1360 | 1.000 | 1.000 | N/A |
